# A method for determining the cutting efficiency of the CRISPR/Cas system in birch and poplar

**DOI:** 10.48130/FR-2021-0016

**Published:** 2021-09-23

**Authors:** Zhibo Wang, Zihang He, Ming Qu, Zhujun Liu, Chao Wang, Yucheng Wang

**Affiliations:** State Key Laboratory of Tree Genetics and Breeding, Northeast Forestry University, 26 Hexing Road, Harbin 150040, China

**Keywords:** *Betula platyphylla*, CRISPR/Cas, cutting efficiency of CRISPR/Cas, transient transformation, *Populus davidiana×P. bolleana*, quantitative PCR

## Abstract

Determination of Cas9 cutting efficiency to the target sites is important for genome editing. However, this determination can only be made via an *in vitro* method, as the purification of Cas protein and synthesis of gRNA are necessary. Here, we developed an *in vivo* method, called transient CRISPR/Cas editing in plants (TCEP) to determine Cas9 cutting efficiency. The CRISPR/Cas vector for plant transformation mediated by *Agrobacterium tumefaciens* was constructed as normal. Using the transient transformation method we built, the Cas9 protein and gRNA were transiently expressed and formed a complex to cut its target sites, resulting in dynamic DNA breakage. The broken DNA was quantified using qPCR to measure the efficiencies of Cas9 cutting. We studied the Cas9 cutting efficiencies to different target sites in *Betula platyphylla* and *Populus davidiana*×*P. bolleana* plants using TCEP and an *in vitro* method. The results of TCEP were consistent with those of the *in vitro* method, suggesting that the TCEP method is reliable in determining cutting efficiency. Additionally, using the TCEP method, we showed that both heat and sonication treatment significantly improved CRISPR/Cas efficiency. Therefore, the TCEP method has broad application value and can not only be used to analyze the CRISPR/Cas efficiency but also to determine the factors involved in Cas9 cutting.

## INTRODUCTION

In recent years, a breakthrough genome editing system has been developed: clustered regularly interspaced short palindromic repeats (CRISPR) and CRISPR-associated (Cas) protein technology. CRISPR-Cas technology is based on a Cas nuclease paired with a guide RNA (gRNA)^[1]^. The CRISPR/Cas system was first discovered in the genomes of many bacteria and most archaea and served as an adaptive immune defense system that protects the host cell by using RNA-guided nucleases to cleave the invasion of nucleic acids such as viral genomes^[2,3]^. Among CRISPR/Cas systems, the CRISPR/Cas9 machinery has been the most studied and widely accepted for its simplicity, robustness, and high efficiency in genome edition^[4]^. The CRISPR/Cas9 system contains two parts: the Cas9 endonuclease and a gRNA. Cas9 nucleases have the conserved domains HNH and RuvC, which have strand-specific cleavage. A gRNA is 20–30 nucleotides (nt) in length usually. The sequence of gRNA is highly gene-specific and facilitates Cas9-mediated precise genome edition by matching complementary nucleic acid sequences. The gRNA is a complementary sequence of target DNA that binds to the target DNA sequence that ends with the protospacer adjacent motif (PAM). For CRISPR–Cas9 from Streptococcus pyogenes, the sequence of PAM is usually 'NGG', which is essential for Cas9 binding and cleavage^[5]^. Adjacent to the 3' end of the 20 nt gRNA is a gRNA scaffold sequence 80 nt in length, which is essential for Cas9 binding. The Cas9 and gRNA proteins first form the gRNA-Cas9 complex and then bind to the target DNA site, and Cas9 performs its nuclease activity to create a double-strand break at the target DNA site exactly 3 bp before the PAM sequence^[6]^. The cutting of the genome will be repaired, which is often accompanied by insertions or deletions in the cleaved site. Therefore, when the cleaved site is in the open reading frame (ORF), it leads to frameshift mutations, interfering with protein translation and thereby disrupting the function of the gene.

CRISPR/Cas9 gene editing technology has been widely used in plant species, including knocking out or knocking down the gene for functional investigation, maintaining heterosis, improving various economic traits, and producing biotic or abiotic stress tolerance plants^[7−10]^. For instance, Wang et al.^[10]^ simultaneously edited meiotic and fertilization genes to combine fixation of heterozygosity, and this strategy finally enabled clonal reproduction of F1 rice hybrids through seeds to maintain heterosis. In improving economic trait studies, Li et al.^[8]^ used CRISPR/Cas9 multitarget gene editing technology to significantly improve the lycopene content in tomato fruit. Rice with knockout of permeable K^+^ transporter OsHAK1 with the CRISPR-Cas system dramatically decreased Cs^+^ uptake, providing perspectives to produce safe food in regions contaminated by nuclear accidents^[11]^. Alfatih et al.^[12]^ generated PARAQUAT TOLERANCE 3 (PQT3) knockout mutants with CRISPR–Cas9 technology. The *OsPQT3* knockout mutants (*ospqt3*) display improved salt stress tolerance and enhanced agronomic performance with higher yield compared with the wild type under salt stress conditions. The Populus knock-out of *4CL1* (4-coumarate: CoA ligase 1) showed lower syringyl-to-guaiacyl (S: G), a reduction in lignin, and an increase in caffeic acid^[13]^.

In biotic and abiotic studies, the *OsERF922* gene was knocked out by using CRISPR/Cas9 in rice, and rice plants displayed substantially improved blast resistance^[14]^. Knockout of EVE (enlarged vessel element) using CRISPR-Cas9 in Populus leads to fewer vessel elements and a reduction in vessel area^[15]^. Mutation of *OsXYN1* (endo-1,4-β-xylanase) in rice with CRISPR-Cas in rice caused thinner stems, less lignin content and reduced water intake^[16]^. Tomato with knockout of LATERAL ORGAN BOUNDARIES DOMAIN (LBD) showed improved water-holding ability and significantly increased drought tolerance^[17]^.

The use of CRISPR-Cas can lead to random vector integrations or the possibility of undesirable genetic alterations caused by plasmid DNA integrating at the cut site, suggesting that a DNA-free gene editing system has been developed. This method did not require the use of DNA vectors and only required RNA and protein components; therefore, there was no incorporation of T-DNA into the host genome^[18]^. DNA-free editing technology has been used in at least 14 plant species^[18]^. The above studies indicated that the CRISPR/Cas system plays an increasingly important role in plant breeding.

However, there were still some problems that restrained the utilization of the CRISPR/Cas system in plants. One of the major concerns for CRISPR/Cas9 editing is the efficiency of editing. The low mutation efficiency in some plants remains a problem^[19]^. However, it is difficult to know whether a certain candidate guide sequence is inefficient or efficient in plants^[20]^. In many studies, the transformed Cas protein cannot cut their target efficiently, causing failure in genome editing. Therefore, selection of the target site that can be cut by Cas protein efficiently is quite important for genome editing. Currently, the cutting efficiency of Cas at DNA sites is mainly determined by an *in vitro* method using purified Cas protein and designed primers to synthesize gRNA *in vitro*, which is time-consuming and complex. In the present study, we developed a simple and robust technology to determine the cutting efficiency *in vivo*.

## RESULTS

### The principle and building of Cas9 cutting using the method of transient CRISPR/Cas editing in plants (TCEP)

**Figure 1:**
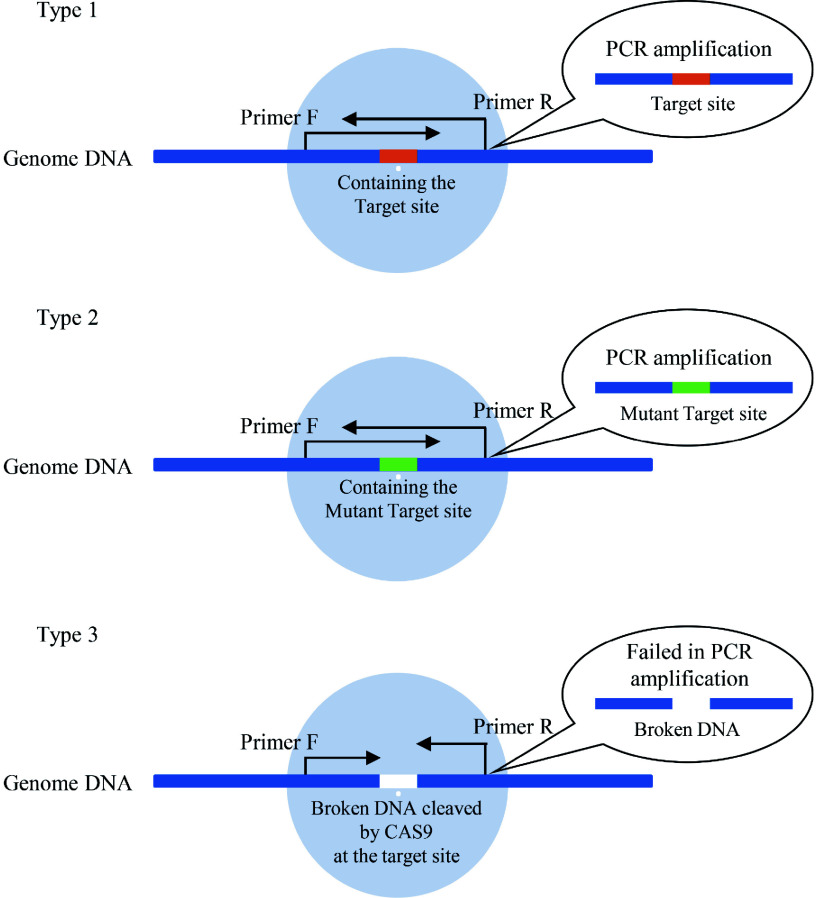
The principle of determination of efficiencies of Cas9 cutting. There are 3 types of DNA present in plant cells after transient transformation of gRNA and Cas9. Type 1: DNA containing the target Cas9 cutting site without breakage (which can be PCR amplified). This kind of DNA has not been cleaved by Cas9 or has been cleaved by Cas9 but has been repaired without a mutant. Type 2: DNA containing the mutant target CRISPR cutting site without breakage (which can be PCR amplified). This kind of DNA had been cut by Cas9 and repaired with mutation. Type 3: the broken DNA by cutting on the target site by CRISPR/Cas. Type 3 DNA cannot be PCR amplified, and its quantities can reflect the efficiency of Cas9 cutting and be detected by qPCR.

Through transient transformation, the genes coding Cas9 and gRNA were both transformed into plant cells, which were transcribed into proteins and gRNA to form the Cas9 protein and gRNA complex to cut off the target DNA site. The CRISPR cutting of DNA will result in broken DNA, causing failure in PCR amplification of this region (Fig. 1). Therefore, if the Cas9 cutting efficiency is high, the amount of broken DNA is correspondingly high (although the repair of cutting DNA is working), causing reduced PCR amplification, which can be detected and quantified by qPCR. After normalizing the expression of Cas9, the cutting efficiency was calculated. In addition, this method can not only detect the cutting efficiency of CRISPR/Cas but can also be used to quickly determine whether the CRISPR/Cas system can work in different plant species.

**Figure 2:**
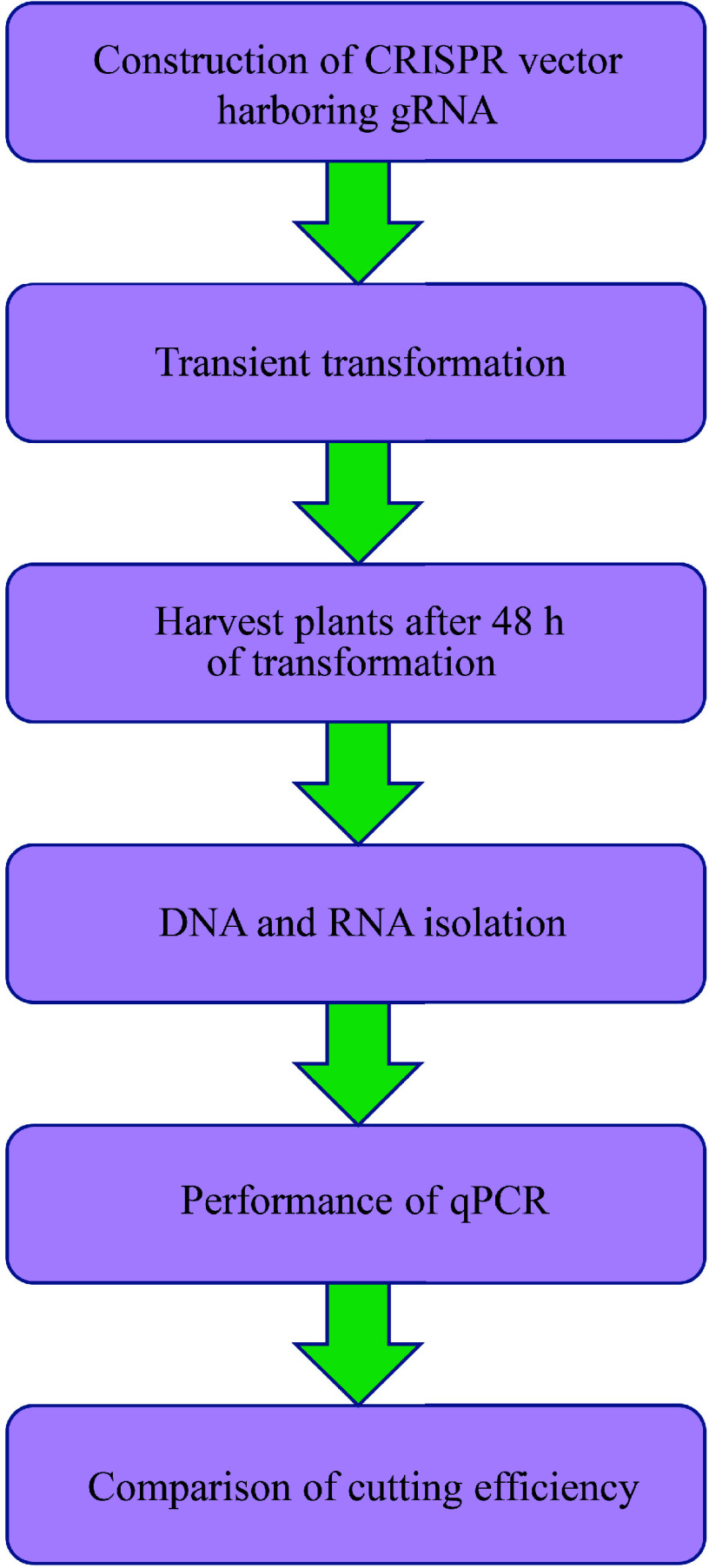
Flowchart of transient CRISPR/Cas editing in plants (TCEP). (1) CRISPR/Cas vectors harboring gRNAs were constructed and transformed into Agrobacterium for plant transformation. (2) Transient transformation was performed on the plants. (3) After transformation for 48 h, the plants were harvested for DNA isolation. (4) Genomic DNA and total RNA were extracted, and RNA was reverse transcribed into cDNA. (5) Quantitative PCR was performed to determine the cutting efficiency, and the cutting efficiency was determined by the amount of broken DNA normalized by the expression of Cas9.

According to the principle above, the procedure for TCEP is shown in Fig. 2. Transient transformation was first performed to transiently express Cas9 and gRNA in plant cells (the detailed transient transformation procedure is shown in the 'Construction of CRISPR/Cas vector and transient genetic transformation' section). After transformation for 48 h, the expression of Cas9 and gRNA reached the peak level (Supplementary Fig. S1), and the samples were harvested to detect cutting efficiency. Genomic DNA was isolated from the samples and used as the template for qPCR. RNA was extracted and reverse transcribed into cDNA and used for analysis of Cas expression. Quantitative PCR was performed to detect the cutting efficiency using primers whose amplification region contained the gRNA sequence (the detailed procedure is shown in the 'Quantitative PCR' section). The cutting efficiency was calculated by the abundance of broken CRISPR product normalized by the abundance of Cas9 transcript (the detailed algorithm was shown in 'The algorithm for calculation of the CRISPR cutting efficiency').

### Determination of the efficiency of CRISPR/Cas mediated by the TCEP method in birch (*Betula platyphylla*)

**Figure 3:**
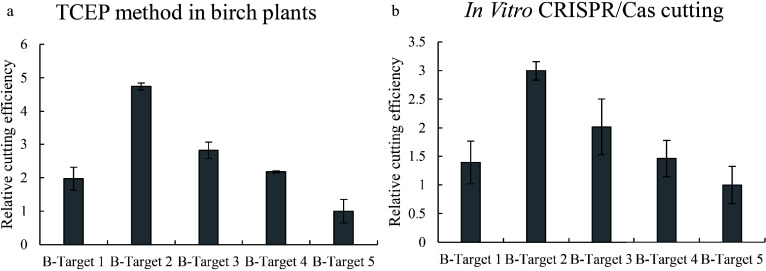
Determination of the efficiencies of Cas9 cutting to different target sites in birch. Five target sites were selected for study, and the efficiencies of Cas9 cutting to different target sites were studied using qPCR. (a): Determination of Cas9 cutting efficiency using the TCEP method in birch. (b): Determination of Cas9 cutting efficiency using the *in vitro* cutting method. Targets 1–5: The CRISPR target cutting sites 1–5 of birch and the sequences of these target sites are shown in Supplemental Table S1. The target site with the lowest cutting efficiency was set as 1 to normalize the efficiencies of Cas9 cutting to other target sites.

After transient transformation for 48 h, we determined the cutting efficiencies of CRISPR/Cas at 5 target sites in birch. After transient transformation, the genome of DNA and total RNA from birch were isolated, and the cutting region and the expression of Cas9 were quantified using qPCR. The qPCR results showed that although Cas9 can work on all these target cutting sites, it has significantly different cutting efficiencies for these target sites (Fig. 3a).

### Verification of TCEP results in birch using the Cas9-mediated *in vitro* cutting method

To further confirm this result, we performed Cas9-mediated cutting *in vitro*. The same target sites were PCR amplified and purified and cut using Cas9 together with gRNA *in vitro*, and qPCR was performed to study the cutting efficiency. The results also showed that these sites can all be cut by Cas9 but showed a significant difference in cutting efficiency (Fig. 3b). In addition, both TCEP and Cas9-mediated cutting *in vitro* displayed very consistent results on cutting efficiency to different target sites (Fig. 3), suggesting that the TCEP method in birch is dependable.

### Determination of the Cas9 cutting efficiency in Shanxin poplar (*Populus davidiana*×*P. bolleana*)

**Figure 4:**
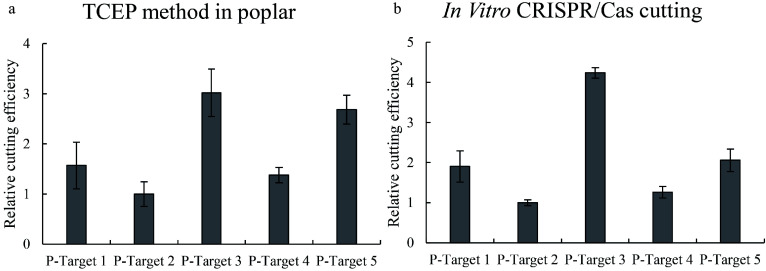
Determination of the efficiencies of Cas9 cutting to different target sites in poplar plants. Five target sites were studied, and the efficiencies of Cas9 cutting to different target sites were determined using qPCR. (a): Determination of Cas9 cutting efficiency using the TCEP method in poplar (*P. davidiana*×*P. bolleana*). (b): Determination of Cas9 cutting efficiency using the *in vitro* cutting method. Targets 1–5: CRISPR target cutting sites 1–5 of poplar, and the sequences of these target sites are shown in Supplemental Table S1. The target site with the lowest cutting efficiency was set as 1 to normalize the efficiencies of Cas9 cutting to other target sites. * indicates a significant difference compared with the control (*p* < 0.05).

The TCEP method was also performed in Shanxin poplar to determine whether it can work well, and 5 target sites were studied. After transient transformation for 48 h, the DNA from each sample was extracted and used for qPCR. The results showed that Cas9 had significantly different cutting efficiencies for these 5 targets (Fig. 4a). The target with the highest Cas cutting activity is more than 6-fold the target with the lowest Cas cutting activity. These results suggested that TCEP can also work well in poplar, and the Cas cutting activities at different target sites are quite different.

### Verification of TCEP results in poplar using the Cas9-mediated *in vitro* cutting method

Cas-mediated cutting *in vitro* was performed to determine the reliability of the TCEP method in poplar. Consistently, Cas9-mediated cutting *in vitro* also showed that the Cas9 protein has different cutting activities at these target sites (Fig. 4b). In addition, both results showed that the cutting efficiencies of Cas to different target sites were consistent (Fig. 4). These results indicated that the Cas9 cutting efficiency determined by TCEP is robust.

### Study of the effect of heat treatment on Cas9 cutting efficiency

**Figure 5:**
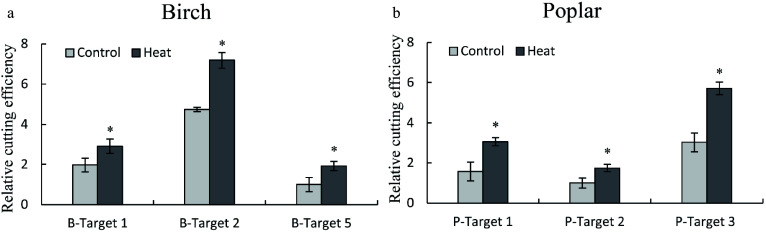
Determination of the effects of heat treatment on the cutting efficiency of CRISPR/Cas using the TCEP method. (a, b): The effects of heat treatment on CRISPR/Cas efficiency in birch (a) and poplar (b). Three target sites with high, medium and low CRISPR cutting efficiency were selected from birch and poplar for study using qPCR. After transient transformation for 48 h, the plants were incubated at 35 °C for 24 h (Heat treatment), and the plants without heat treatment were harvested at the same time as the control (Control). The serial numbers of target cutting sites of birch and poplar are consistent with Fig. 2 and Fig. 3, respectively. The target site with the lowest cutting efficiency was set as 1 to normalize the efficiencies of Cas9 cutting to other target sites. * indicates a significant difference relative to the control (*P* < 0.05).

LeBlanc et al.^[21]^ showed that heat treatment can significantly improve Cas9 cutting. To verify the effect of heat treatment on Cas9 cutting, TCEP was used. After transient transformation for 48 h, the plants were incubated at 35 °C for 24 h, and the transiently transformed plants under normal conditions were used as the control. After heat treatment, genomic DNA was isolated from birch or poplar and used as the template for qPCR. The effects of heat treatment on Cas9 cutting were determined using qPCR by amplification of the truncated DNA containing target sites. The results showed that 35 °C treatment significantly improved the cutting efficiencies at the target site in both birch (Fig. 5a) and poplar plants (Fig. 5b), suggesting that heat treatment can increase the cleavage activities of CRISPR/Cas. This result is consistent with previous results determined using *Arabidopsis thaliana* with stable transformation of CRISPR/Cas^[21]^.

### Sonication can improve CRISPR-Cas cutting ability by increasing genetic transformation efficiency

**Figure 6:**
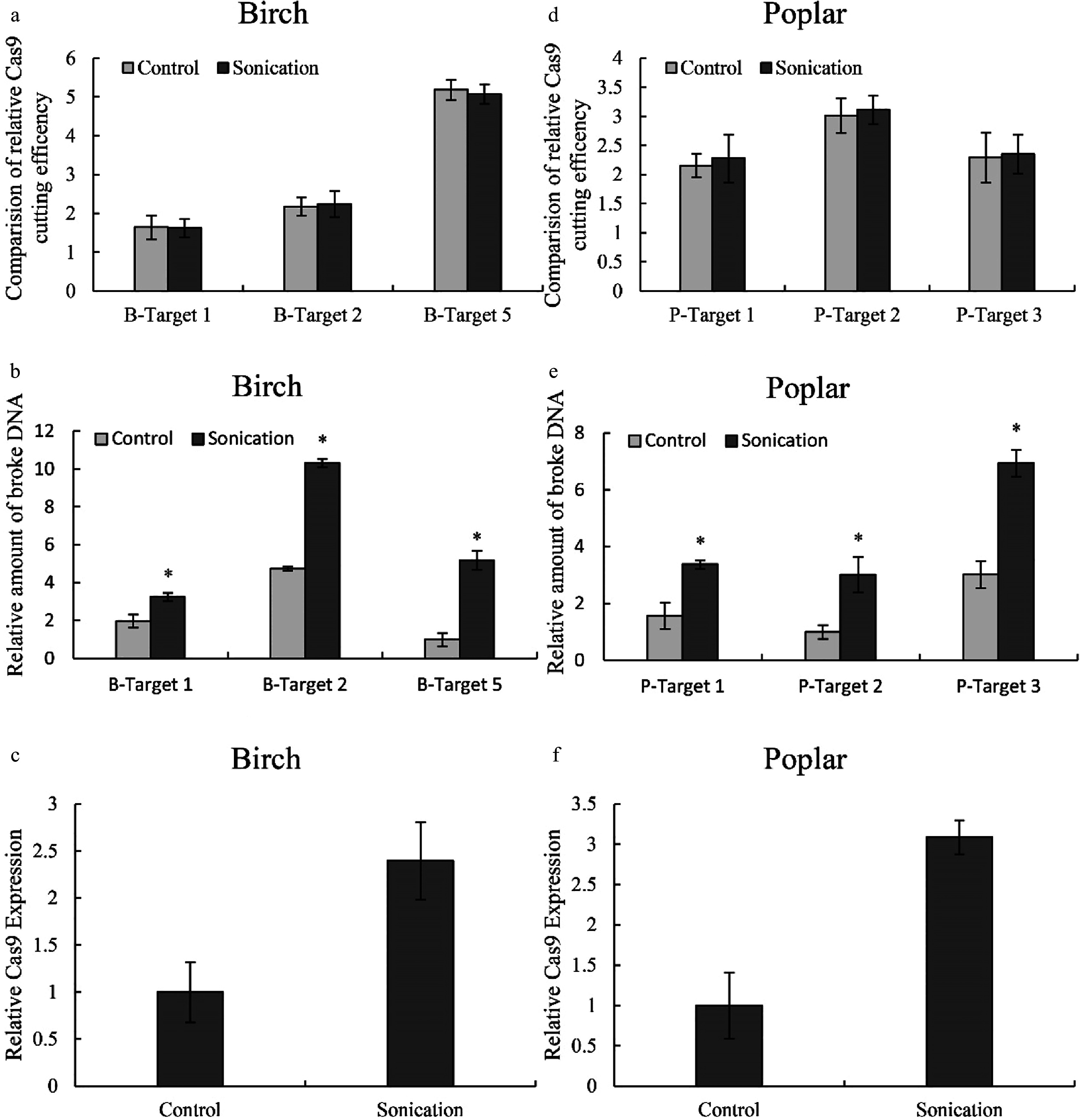
Determination of the effects of sonication on Cas9 cutting efficiency. (a, d): The effects of sonication on Cas9 cutting efficiency in birch (a) or poplar (d); (b, e): The relative amount of broken DNA in birch (b) or poplar (e) with or without sonication treatment. (c, f): Study of the expression of *Cas9* in birch (c) or poplar (f) with or without sonication treatment. The plants were sonicated for 10 seconds, and the TCEP method was performed (Sonication). Plants without sonication were used as controls (Control). The number of target cutting sites of birch and poplar is consistent with Fig. 2 and Fig. 3, respectively. The target site with the lowest cutting efficiency or amount of broken DNA was set as 1 for normalization Cas9 cutting. Three target sites with high, medium and low CRISPR cutting efficiency were selected for study using qPCR. (c, f): The expression of the gene encoding *Cas9* with or without sonication treatment. * indicates a significant difference compared with the control (*p* < 0.05).

As sonication can improve genetic transformation, we also studied whether this treatment could increase CRISPR-Cas efficiency in both birch and Shanxin poplar. Three target cutting regions from birch and Shanxin poplar that showed high, medium and low cutting efficiency were studied. After normalization by the expression of Cas9, the cutting efficiency was not significantly altered after sonication (Fig. 6a & 6d). However, the amount of broken DNA was significantly increased in both birch (Fig. 6b) and poplar (Fig. 6e). Additionally, the transcript level of the *Cas9* gene was improved correspondingly in both birch (Fig. 6c) and poplar (Fig. 6f). Therefore, sonication can improve Cas9 cutting by increasing the expression of *Cas9* and gRNA.

## DISCUSSION

The CRISPR/Cas system is the most important gene-editing technology that has been used in a variety of basic biological studies, diagnosis and management of diseases and has broad application prospects^[22,23]^. Therefore, intensive study, optimization and expansion of the application of the CRISPR/Cas system are important and necessary. In the present study, we proposed a TCEP method for quick determination of the cutting efficiency of CRISPR/Cas, which will be helpful in the generation of genome editing plants with CRISPR/Cas and used in a broad aspect.

### Quantitative PCR can be used to determine the Cas9 cutting efficiency

During the period of *Cas* gene and gRNA transient expression in plant cells, there are 3 types of DNA in cells, i.e., DNA containing the target site but without breakage, DNA with the mutant target region without breakage, and DNA broken in the target site by the Cas9/gRNA complex (Fig. 1). In addition, the broken DNA will exist dynamically because the Cas9/gRNA complex will continually cut the DNA containing the target region in the cells, which will cause broken DNA cleavage in the target region. In addition, although some target regions had been cut off, they were repaired without mistake (i.e., no resulting mutant), and will be cutting again. As the broken DNA cannot be PCR amplified when the PCR amplified region contains the breaking site, it can be detected and quantified by qPCR to evaluate the efficiencies of Cas9 cutting. In the present study, our results confirmed this hypothesis.

The qPCR results clearly indicated that many broken DNA existed during transient transformation (Fig. 3 & Fig. 4), and the broken DNA was increased when the expression of Cas9 increased (Fig. 6). The quantities of broken DNA can reflect the CRISPR/Cas cutting efficiencies to different target regions because the results of TCEP are consistent with those of Cas9 cutting *in vitro* (Fig. 3 & Fig. 4). In addition, our results showed that compared with the plants without Cas9 cutting, the highest ΔΔCt can reach nearly 2, suggesting that the highest percentage of broken DNA can reach nearly 75% of total DNA during transient transformation. Of course, the editing efficiency not only relies on the cutting efficiency of gRNA and the Cas9 complex but also depends on the DNA repair apparatus in a given cell type; thus, the evaluation of the DNA cutting efficiency may not exactly reflect the editing efficiency. However, as repair of the broken DNA may lead to the mutation of DNA with a large probability, qPCR results can be used to determine the probability of the mutation mediated by CRISPR/Cas.

The limitation of the TCEP method concerned may be that the mutated sequence mediated by CRISPR cannot be detected in the TCEP method. However, this limitation might not significantly influence the accuracy of TCEP for the following two reasons. One reason is that broken DNA cannot exist for a long time and will be repaired soon; therefore, the TCEP method only determines the broken DNA at one time point. However, the mutant sequence (Type 2) is generated not at one time point but for a period of time since the cutting of Cas. Taking into account the mutant sequence (Type 2) will affect the accuracy of the cutting result because TCEP only detects the situation of cells in a short time. Therefore, the dynamic broken DNA could generally reflect the Cas cutting efficiency during the period of time detected by the TCEP method. The other reason is that the type 2 DNA (mutated sequence) was discarded in all the studied samples in the determination of cutting efficiency, which is fair to these samples when calculating the relative cutting efficiency. Therefore, only calculation of the broken DNA could also reflect the relative efficiency of cutting. In addition, the *in vitro* cutting method was consistent with the results of TCEP (Fig. 3b; Fig. 4b), confirming that only the detection of broken DNA to evaluate the relative cutting efficiency of Cas is reliable.

### The usage of TCEP method in CRISPR/Cas study

TCEP can be used to detect the CRISPR cutting efficiencies in all plants that can be infected with *Agrobacterium tumefaciens* and is not dependent on whether the transformation system is stable. Compared with the *in vitro* Cas9 cutting method, this method does not require purification of the Cas9 protein, synthesis of gRNA *in vitro* or preparation of the DNA template; therefore, this method has the advantages of simplicity.

In addition to detection of the efficiency of Cas9 cutting to different target sites, the TCEP method can also be used in optimization of the factors involved in Cas9 cutting. For instance, previous studies showed that heat treatment can increase the cutting efficiency of Cas9^[21]^. However, these results were obtained from stably transformed plants, which will take a long time and require much work. In this study, we used the TCEP method to determine whether heat treatment can increase Cas9 cutting efficiency, and the results showed that heat treatment can improve the Cas9 cutting efficiency (Fig. 5), which is consistent with the study of LeBlanc et al^[21]^. In addition, using the TCEP study, our results showed that treatment with sonication can significantly increase the efficiency of Cas9 cutting (Fig. 6). Furthermore, the efficiencies of Cas9 cutting can be calculated quantitatively. Therefore, the TCEP method can be used to optimize CRISPR editing by determining the factors involved in Cas9 cutting efficiency and has good application in the future.

To date, many plant species do not have a genetic transformation system and thus do not know whether the CRISPR/Cas system can work well in these plant species. Our method will provide a solution strategy, and the work efficiency of CRISPR/Cas will be determined quantitatively in all plant species that can be transformed by *A. tumefaciens* using the TCEP method. Therefore, the TCEP method will also improve the predictability of CRISPR/Cas studies.

## CONCLUSIONS

We developed a method called TCEP, which can determine the cutting efficiencies of CRISPR/Cas to different target sites and has the advantages of robustness and simplicity. In addition, the TCEP method can also be used to optimize the CRISPR/Cas system because it can perform CRISPR/Cas analysis not dependent on stable transformation and can be completed within 3–4 days. This method may be suitable for a variety of plants. In theory, it can be used in any plant species that can be transformed by Agrobacterium. Therefore, this method has broad applications in CRISPR/Cas studies.

## MATERISALS AND METHODS

### Plant materials

Birch (*B. platyphylla*) and Shanxin poplar (*Populus. davidiana×P. bolleana*) were tissue culture plantlets that were grown in a tissue culture room with a 10 h light/14 h dark photocycle, 75% relative humidity, and a stable temperature of 25 °C. The birch plantlets were grown in solid culture medium (WPM + 1 mg·L^−1^ 6-BA + 20 g·L^−1^ sucrose, pH 5.8). Plantlets of Shanxin poplar were grown on solid culture medium (1/2 MS + NAA 0.05 mg/L + 6-BA 0.5 mg/L).

### Construction of the CRISPR/Cas vector and transient genetic transformation

The gRNA was designed using the online program (http://skl.scau.edu.cn), 5 sites from the birch genome^[24]^ and Shanxin poplar genome (unpublished) were selected according to the predicted cutting efficiency, and the sites with predicted high, medium and low cutting efficiency were selected for study. The primer pairs for gRNA were synthesized and cloned into the pYLCRISPR/Cas9P_35S_-N vector to construct a paired-sgRNA/Cas9 binary^[25]^ and transformed into *A. tumefaciens* EHA105.

Transient genetic transformation was performed according to Zang et al.^[26]^. The plants (birch or Shanxin poplar) were incubated in transformation buffer [2 mM Mes-KOH (pH 5.8), 2% (w/v) sucrose, 270 mM mannitol, 120 μM acetosyringone, 40 mM CaCl_2,_ 20 μM 5-azacytidine, 200 mg/L DTT + 0.7 OD600 *A. tumefaciens*, 0.02% (w/v) Tween 20], and shaken at 100 rpm for 1.5 h at 25 °C. Then, the plants were quickly washed with sterilized water to remove excess *A. tumefaciens* and cultured in solid medium [MS, 2% (w/v) sucrose, 150 μM acetosyringone, 200 mg/L DTT, pH 5.4] for 48 hours. After transformation, DNA was isolated from the transgenic plants, and quantitative PCR (qPCR) was performed.

### The *in vitro* cutting of CRISPR

To determine the cutting efficiency of Cas9 *in vitro*, a Cas enzyme *in vitro* digestion kit (P1400, Invogen Tech. Co, China) was used. gRNA was synthesized using an sgRNA transcription kit (PC1380) according to the protocol (Inovogen, Beijing, China). The gRNA, purified Cas9 protein and target DNA were incubated together at 37 °C for 50 min. After incubation, the products were purified using a PCR purification kit (Qiagen*,* catalog *# 28104*) and used for qPCR.

### Quantitative PCR

Total DNA was isolated from birch and Shanxin poplar using a DNA isolation kit (DP350) from Tiangen Biotech Co., Ltd. (Beijing, China). Total RNA was isolated from birch or Shanxin poplar using the PLANTeasy RNA isolation kit (BioTeke, Beijing, China) and digested using DNase I (RNase free). Total RNA was reverse transcribed into cDNA with oligo(dT) primers using a PrimeScript™ RT reagent Kit (TaKaRa, Kaiseki Kiyomoto, Japan), diluted with ultrapure water to 100 µl, and used as PCR templates. Real-time PCR or quantitative real-time PCR (qRT–PCR) was carried out as follows: a 20 µl reaction volume containing 0.5 µM of each forward or reverse primer, 10 µl of SYBR Premix Ex Taq™ (TaKaRa), and 2 µl of cDNA as the PCR template. Real-time PCR was carried out on a qTower 2.2 system (Analytik Jena AG, Jena, Germany). The thermal profile was 94 °C for 30 s; then, 45 cycles of 94 °C for 12 s, 58 °C for 30 s, and 72 °C for 45 s were performed.

### The algorithm for calculation of the CRISPR cutting efficiency

To determine the Cas9 cutting efficiencies of the different DNA sites *in vivo*, transient transformation was performed, and *ubiquitin* was used as the internal reference (which can be used in both qPCR and qRT–PCR, whose primers are shown in Supplemental Table S1). The relative cutting efficiency was determined by the abundance of broken DNA divided by the abundance of Cas9 expression. The abundance of the digest CRISPR product was calculated as 2^–[(Ct(T)-Ct(T-inter)-Ct(C)+Ct(C-inter)]^, where Ct(C) and Ct(C-inter) indicate the Ct value of PCR amplification of the target site region and the internal reference in the control plants (the control plants refer to WT plants without any treatment, whose DNA was used as the template for qPCR), respectively. Ct(T) and Ct(T-inter) indicate the Ct values of PCR amplification of the target site region and PCR amplification of the internal reference in the test plants, respectively (test plants refer to the CRISPR/Cas editing plant, whose DNA was used as the template for qPCR). To determine the expression of Cas9, qRT–PCR was performed, and the internal reference *ubiquitin* was used to normalize its expression. The abundance of Cas9 expression was determined by 2^–^^[Ct(Cas)-Ct(inter)]^, where Ct(Cas) indicates the Ct value of expression of Cas9 and Ct(inter) indicates the Ct value of expression of internal reference gene in the CRISPR/Cas editing plants. The relative cutting efficiency was determined as 2^–[(Ct(T)-Ct(T-inter)-Ct(C)+Ct(C-inter)]^/2^–[Ct(Cas)-Ct(inter)]^. Three biological replications were performed.

For analysis of *in vitro* Cas9 cutting efficiency, after incubation, the DNA products were purified and used for qPCR. qPCR was performed to determine the cutting efficiency of different regions, which was calculated as the quantity of digested DNA divided by the quantity of total DNA. The cutting efficiency was calculated as 1–1/2^[Ct(a)-Ct(inter)]^, where Ct(a) indicates the Ct value of PCR amplification of truncated DNA containing the target site, and Ct(inter) refers to the Ct value of PCR amplification of truncated DNA without a target site (which calculates the total DNA).

### Statistical analyses

Statistical analyses were carried out using SPSS 16.0 (IBM Corp., Armonk, NY, USA) software. Data were compared using Student's t-test. Differences were considered to be significant if *p* < 0.05. * represents *p* < 0.05.

## SUPPLEMENTARY DATA

Supplementary data to this article can be found online.
