# Supplementary material for: A method for determining the cutting efficiency of the CRISPR/Cas system in birch and poplar
Source: For Res (Fayettev). 2021 Sep 23;1:16. doi: 10.48130/FR-2021-0016 (PMC11524279; doi:10.48130/FR-2021-0016)
Supplement: Supplementary file 1 — Supplementary data to this article can be found online. [file FR-2021-0016-S1.zip › 10.48130_FR-2021-0016-Suppl-TableS1.docx]

Supplemental Table 1 The primer sequences used for genes studied in real-time PCR and vector construction

| Gene name | Primer sequences (5’-3’);  F: Forward; R: Reverse |
| --- | --- |
| Tubulin | F: TCAACCGCCTTGTCTCTCAGG  R: GGTTGTCACGTAAGCTCGGT |
| ACTIN | F: GCTGAGAGATTCCGTTGCCCTG  R: GGCGGTGATCTCCTTGCTCATT |
| BpTarget1,2 | F: CAACTGAAGAAGAGCTGGT  R: TCCTCTAGCTTCTCTTTCTTG |
| BpTarget3,4 | F: TTCCGTGCATTTGATCGACGC  R: TTATCTCCCAGGTAATGCTTC |
| BpTarget5 | F: TCAGGAGAGCGGTGTAAAG  R: GCCTTCCCCTTATAGAAAAC |
| PbTarget1,2,3,5 | F: TGCGTGGTTACCAATCCC  R: CAATCAACAATCAGCGCTCC |
| PbTarget4 | F: GCTGGATTAACCAATCCTAAACAC  R: CCTGTTGCTTGGGACTGCC |
| Cas9 | F: ACCTTCGTAAGAAGCTTG  R: CTCGAGACGCCTGGACTTG |

| Gene name | Primer sequences (5’-3’);  F: Forward; R: Reverse |
| --- | --- |
| B-Target1 | F: GAAGAGACCAGAAATCCAAGTTTTAGAGCTAGAAAT  R: TTGGATTTCTGGTCTCTTCCAATCTCTTAGTCGACT |
| B-Target2 | F: TAAAGCGGACACTGAGCAAGTTTTAGAGCTAGAAAT  R: TTGCTCAGTGTCCGCTTTACAATCTCTTAGTCGACT |
| B-Target3 | F: AACATCCCAGTGAGAGGGCGTTTTAGAGCTAGAAAT  R: GCCCTCTCACTGGGATGTTCAATCTCTTAGTCGACT |
| B-Target4 | F: TCTCAGAATGCTACAACGGGTTTTAGAGCTAGAAAT  R: CCGTTGTAGCATTCTGAGACAATCTCTTAGTCGACT |
| B-Target5 | F: AACTACAGTTTCTGGGTACGTTTTAGAGCTAGAAAT  R: GTACCCAGAAACTGTAGTTCAATCTCTTAGTCGACT |
| P-Target1 | F: CAAGAATTCATCATGATGCGTTTTAGAGCTAGAAAT  R: GCATCATGATGAATTCTTGCAATCTCTTAGTCGACT |
| P-Target2 | F: AGAAGCCTGCTTCTCCAACGTTTTAGAGCTAGAAAT  R: GTTGGAGAAGCAGGCTTCTCAATCTCTTAGTCGACT |
| P-Target3 | F: GGAGCGGTTTGTGGTGATAGTTTTAGAGCTAGAAAT  R: TATCACCACAAACCGCTCCCAATCTCTTAGTCGACT |
| P-Target4 | F: AGTTCCGATTCACCTTATTGTTTTAGAGCTAGAAAT  R: AATAAGGTGAATCGGAACTCAATCTCTTAGTCGACT |
| P-Target5 | F: GTTTTGTTTCTGTTATCGTGTTTTAGAGCTAGAAAT  R: ACGATAACAGAAACAAAACCAATCTCTTAGTCGACT |
|  |  |

Primer sequences for real-time PCR

Primer sequences for vector construction
