# Supplementary material for: A method for determining the cutting efficiency of the CRISPR/Cas system in birch and poplar
Source: For Res (Fayettev). 2021 Sep 23;1:16. doi: 10.48130/FR-2021-0016 (PMC11524279; doi:10.48130/FR-2021-0016)

**Supplementary Figure**

Supplementary Figure 1 The expression of *Cas9* in different transient transformation time point.


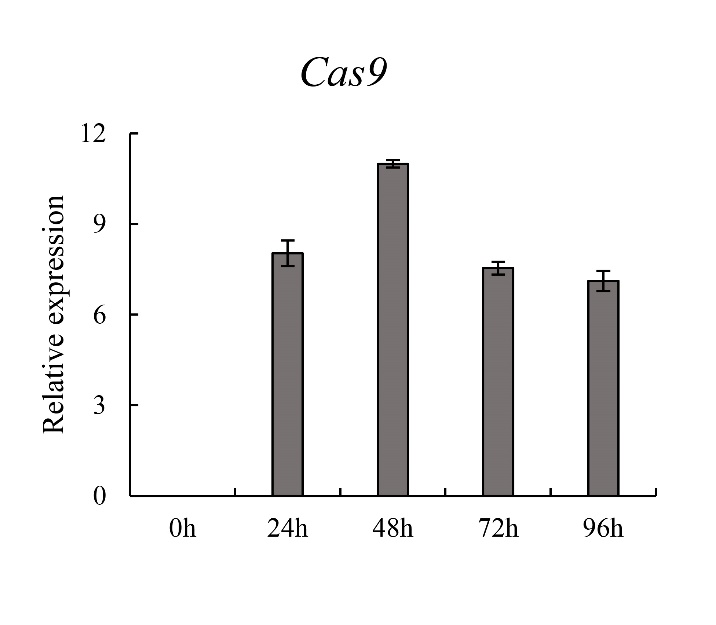

Supplement: Supplementary file 1 — Supplementary data to this article can be found online. [file FR-2021-0016-S1.zip › 10.48130_FR-2021-0016-Suppl-FigureS1.docx]
